# Supplementary figures and images for: A saposin deficiency model in Drosophila: Lysosomal storage, progressive neurodegeneration and sensory physiological decline
Source: Neurobiol Dis. 2017 Feb;98:77–87. doi: 10.1016/j.nbd.2016.11.012 (PMC5319729; doi:10.1016/j.nbd.2016.11.012)

**A***dSap-r*<sup>NP7456</sup>

+/+

mCD8eGFP

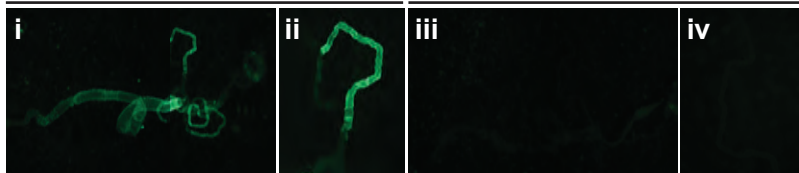

DAPI

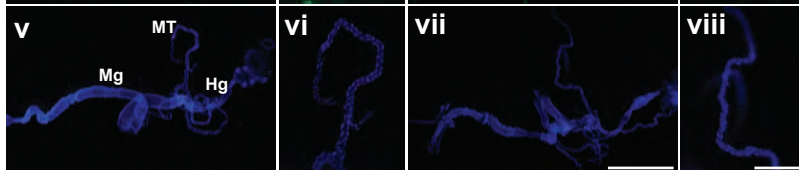**C***dSap-r*<sup>NP7456</sup>

+/+

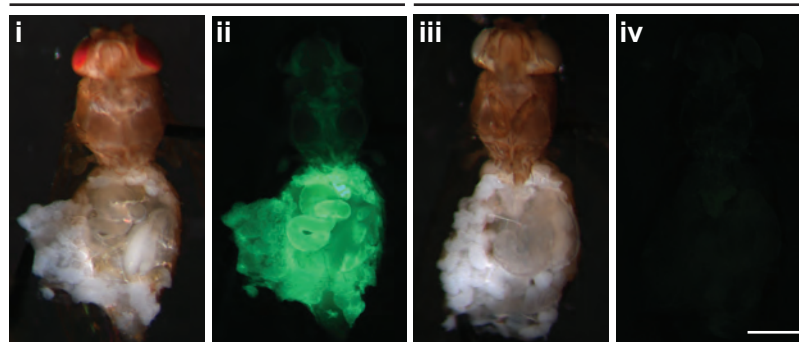**B***dSap-r*<sup>NP7456</sup>

+/+

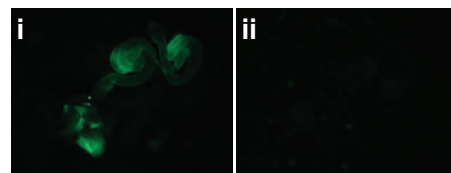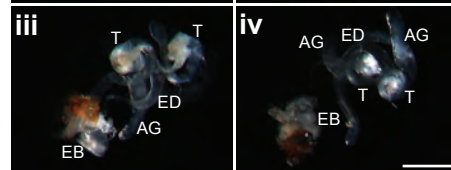**D***dSap-r*<sup>NP7456</sup>

+/+

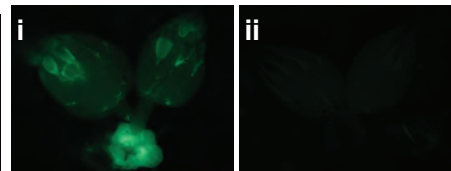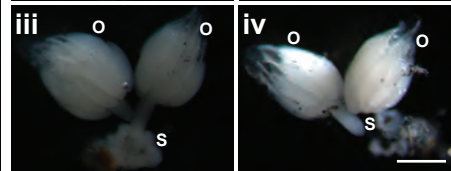

Supplement: Supplemental Fig. 1 — dSap-r is expressed in visceral organs of Drosophila. Digestive systems (A), male (B) and female (D) reproductive systems and fat bodies (C) are shown from adult controls (+/+) and flies expressing mCD8eGFP under the control of dSap-rNP7456 GAL4. Organs are stained with the nuclear marker DAPI in (A). MT, Malpighian tubule; Mg, midgut; Hg, hindgut; T, testes; EB, ejaculatory bulb; AG, accessory gland; ED, ejaculatory duct; O, ovary; S, spermatheca. Scale bars: (A) 1000 μm (vii) and 250 μm (viii), (B-D) 500 μm. [file mmc1.pdf]

**+/+**

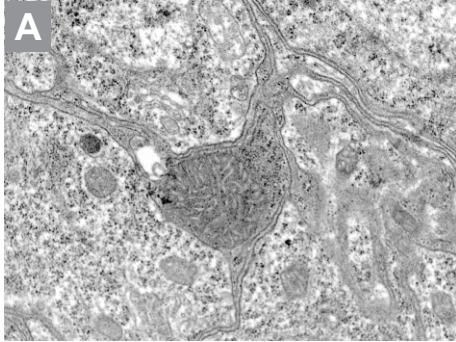

***dSap-r<sup>C27</sup>/Df***

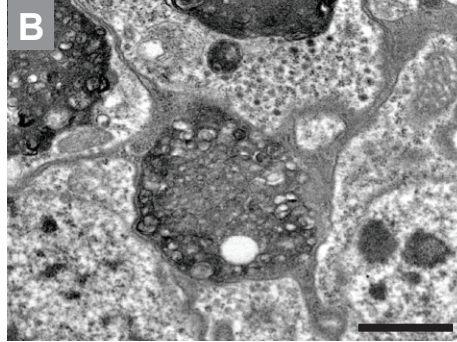

Supplement: Supplemental Fig. 2 — Cell enlargement and increased storage in dSap-rC27/Df mutant glia. Transmission electron micrographs of glia surrounding the antennal lobe of 22-day old wild type (+/+; A) and dSap-rC27/Df mutant (B) brains (n = 3). Electron-dense and electron-lucent vesicular storage is shown in dSap-rC27/Df mutant glia (B). Scale bar: 1 μm. [file mmc2.pdf]
