# Supplementary material for: A saposin deficiency model in Drosophila: Lysosomal storage, progressive neurodegeneration and sensory physiological decline
Source: Neurobiol Dis. 2017 Feb;98:77–87. doi: 10.1016/j.nbd.2016.11.012 (PMC5319729; doi:10.1016/j.nbd.2016.11.012)
Supplement: Supplemental Table 1 — Internal Standard (IS) mix used for lipidomics [file mmc3.pdf]

Supplemental Fig. 1

dSap-r* is expressed in visceral organs of *Drosophila. Digestive systems (A), male (B) and female (D) reproductive systems and fat bodies (C) are shown from adult controls (+/+) and flies expressing mCD8eGFP under the control of *dSap-r*^{NP7456} GAL4. Organs are stained with the nuclear marker DAPI in (A). MT, Malpighian tubule; Mg, midgut; Hg, hindgut; T, testes; EB, ejaculatory bulb; AG, accessory gland; ED, ejaculatory duct; O, ovary; S, spermatheca. Scale bars: (A) 1000 μm (vii) and 250 μm (viii), (B-D) 500 μm.

Supplemental Fig. 2

Cell enlargement and increased storage in *dSap-r*^{C27}/Df mutant glia.
Transmission electron micrographs of glia surrounding the antennal lobe of 22-day old wild type (+/+; A) and *dSap-r*^{C27}/Df mutant (B) brains (n = 3). Electron-dense and electron-lucent vesicular storage is shown in *dSap-r*^{C27}/Df mutant glia (B). Scale bar: 1 μm.

Supplemental Table 1: Internal Standard (IS) mix used for lipidomics

LIPID STANDARD	Catalog No.	SOURCE	Amount added to extract (pmol)
CerPE-C12 Sphingosyl PE [d17:1]	110753	Avanti Polar Lipids, USA	7.96
PC [17:0-14:1]	LM-1004		4.43
PE [17:1-14:0]	LM1104		4.29
C17-Cer [d18:1/17:0]	860517P		10.41
PE-OO [40:00]	999985P		7.16
Sphingosine [d17:1]	LM-2000		7.1
PI [17:0-14:1]	LM-1504		3.72
GluCer[d18:1/12:0]	860543P		7.89
PS [17:0-14:1]	LM-11304		2.95
